# Comparison of quantitative whole body PET parameters on [^68^Ga]Ga-PSMA-11 PET/CT using ordered Subset Expectation Maximization (OSEM) vs. bayesian penalized likelihood (BPL) reconstruction algorithms in men with metastatic castration-resistant prostate cancer

**DOI:** 10.1186/s40644-024-00702-x

**Published:** 2024-05-06

**Authors:** Narjess Ayati, Lachlan McIntosh, James Buteau, Ramin Alipour, Michal Pudis, Nicholas Daw, Price Jackson, Michael S. Hofman

**Affiliations:** 1 Prostate Cancer Theranostics and Imaging Centre of Excellence, Molecular Imaging and Therapeutic Nuclear Medicine, Cancer Imaging, Peter MacCallum Cancer Centre, Melbourne, VIC Australia; 2 Department of Theranostics and Nuclear Medicine, St. Vincent’s Hospital, Sydney, NSW Australia; 3 Department of Physical Sciences, Peter MacCallum Cancer Centre, Melbourne, Australia; 4 Sir Peter MacCallum Department of Oncology, University of Melbourne, Melbourne, VIC Australia; 5 Department of Nuclear Medicine, Bellvitge University Hospital, Barcelona, Spain

**Keywords:** PSMA PET/CT, Metastatic castration-resistant prostate cancer (mCRPC), Bayesian penalized likelihood (BPL), Ordered subset expectation maximization (OSEM), Image reconstruction

## Abstract

**Background:**

PSMA PET/CT is a predictive and prognostic biomarker for determining response to [^177^Lu]Lu-PSMA-617 in patients with metastatic castration resistant prostate cancer (mCRPC). Thresholds defined to date may not be generalizable to newer image reconstruction algorithms. Bayesian penalized likelihood (BPL) reconstruction algorithm is a novel reconstruction algorithm that may improve contrast whilst preventing introduction of image noise. The aim of this study is to compare the quantitative parameters obtained using BPL and the Ordered Subset Expectation Maximization (OSEM) reconstruction algorithms.

**Methods:**

Fifty consecutive patients with mCRPC who underwent [^68^Ga]Ga-PSMA-11 PET/CT using OSEM reconstruction to assess suitability for [^177^Lu]Lu-PSMA-617 therapy were selected. BPL algorithm was then used retrospectively to reconstruct the same PET raw data. Quantitative and volumetric measurements such as tumour standardised uptake value (SUV)max, SUVmean and Molecular Tumour Volume (MTV-PSMA) were calculated on both reconstruction methods. Results were compared (Bland-Altman, Pearson correlation coefficient) including subgroups with low and high-volume disease burdens (MTV-PSMA cut-off 40 mL).

**Results:**

The SUVmax and SUVmean were higher, and MTV-PSMA was lower in the BPL reconstructed images compared to the OSEM group, with a mean difference of 8.4 (17.5%), 0.7 (8.2%) and − 21.5 mL (-3.4%), respectively. There was a strong correlation between the calculated SUVmax, SUVmean, and MTV-PSMA values in the OSEM and BPL reconstructed images (Pearson r values of 0.98, 0.99, and 1.0, respectively). No patients were reclassified from low to high volume disease or vice versa when switching from OSEM to BPL reconstruction.

**Conclusions:**

[^68^Ga]Ga-PSMA-11 PET/CT quantitative and volumetric parameters produced by BPL and OSEM reconstruction methods are strongly correlated. Differences are proportional and small for SUVmean, which is used as a predictive biomarker. Our study suggests that both reconstruction methods are acceptable without clinical impact on quantitative or volumetric findings. For longitudinal comparison, committing to the same reconstruction method would be preferred to ensure consistency.

**Supplementary Information:**

The online version contains supplementary material available at 10.1186/s40644-024-00702-x.

## Background

Following the positive results of the TheraP [1] and VISION [2] trials, [^177^Lu]Lu-PSMA targeted radionuclide therapy has been established as new personalized standard-of-care cancer treatment for patients with metastatic castration-resistance prostate cancer (mCRPC). PSMA PET/CT is as a companion imaging tool for selecting patients suitable for and who may benefit from subsequent ^177^Lu-PSMA therapy. Using quantification, PSMA PET/CT can provide additional predictive and prognostic data with critical guidance on treatment selection in mCRPC [3, 4]. However, quantitative parameters need to be standardized prior to application as biomarkers.

One of the important influencing factors for calculated quantitative parameters is the applied reconstruction method in image processing. The most common used PET image reconstruction algorithm is the ordered subset expectation maximization (OSEM), which is an iterative statistical algorithm [5]. As the number of iterations increase, the noise will also increase, reducing the image quality. This was partially circumvented with the introduction of time-of-flight (TOF)-based systems which can reach to total activity convergence at low iterations [6, 7].

Bayesian penalized likelihood (BPL) reconstruction algorithm, also known as Q.Clear by General Electric (GE Healthcare, Milwaukee, WI, USA), is a relatively newer reconstruction algorithm that improves spatial resolution due to its convergence [8]. It utilizes an iterative function with an added regularization function. The core advantage of this activity-dependent noise control function, also known as relative difference penalty, is its ability to de-noise the image while preserving the edges and achieving full convergence compared to OSEM with partial convergence. Q.Clear also uses a beta factor which controls the relative strength of the regularization. Moreover, point-spread function (PSF) is counted inside the Q.Clear model which improves the spatial resolution [9–12]. However, variations in image processing methods may result in inconsistent quantification of common PET biomarkers across sites or equipment.

Our retrospective analysis aimed to evaluate the impact of using different reconstruction algorithms on the produced quantitative parameters in the same set of patients undergoing [^68^Ga]Ga-PSMA-11 PET/CT.

## Methods

The study population consisted of consecutive patients with mCRPC undergoing [^68^Ga]Ga-PSMA-11 PET/CT at Peter MacCallum Cancer Centre as workup for [^177^Lu]Lu-PSMA-617 therapy from July 2022. Based on a previous study on ^18^F-PSMA-1007 [13], we expected a mean difference of 10% +/- 20% in SUV metrics between Q.Clear and OSEM reconstructed images. From a power calculation of Power = 0.8, with a significance set to 5%, we estimated a minimum required sample size of 34. Therefore, we intended to include 50 patients. To be eligible for the study, patients had to meet the following eligibility criteria: age > 18-year-old, having a diagnosis of mCRPC and undergoing a [^68^Ga]Ga-PSMA-11 PET/CT as work up for [^177^Lu]Lu-PSMA-617 therapy being performed in a prospective registry (NCT04769817). This retrospective study was approved by the Peter MacCallum Cancer Centre Human Ethics & Governance committee (Approval number: HREA/91,235/PMCC) and the committee waved the requirement to obtain patient consent.

Patients were scanned as their routine clinical workup, using one of three GE Discovery PET/CT scanners of the same generation (GE 690 and GE 710), both with LYSO crystal, 15.7 cm axial FOV and 18 min average of scanning duration. Demographics, clinicopathologic, and treatment information were collected. The images were reconstructed once as being done routinely using GE-VPFXS algorithm (OSEM incorporating time-of-flight and resolution recovery using 2 iterations and 24 subsets, matrix size:192 × 192, Gaussian filter: 6.4 mm) and then by BPL algorithm (Q.Clear with a Beta value of 600 and same matrix size of 192 × 192) [8]. Subsequently, the images were processed by a nuclear medicine doctor experienced in [^68^Ga]Ga-PSMA-11 image processing using MIM version 7.1 (MIM software, Beachwood, OH, USA), and the standardised uptake value (SUV)max, SUVmean and Molecular Tumour Volume (MTV-PSMA) were calculated in each set of reconstructed images separately. A lower threshold of SUV 3 was used for calculating MTV with a semi-automated workflow. A second nuclear medicine specialist reviewed processed images to ensure that the contouring is optimal.

To compare the absolute SUVmax, SUVmean and MTV-PSMA by each reconstruction method (BPL vs. OSEM), Bland-Altman plot and statistics were used to describe the mean of differences between the two measurements (with 95% confidence intervals). A difference > 10% in SUVmean was considered significant. To compare correlation between quantitative parameters, a scatter plot was generated and Pearson’s correlation co-efficient calculated for SUVmax, SUVmean and MTV-PSMA. A two by two table with SUVmean ≥ 10 and SUVmean < 10 was used to describe how patients change category following Q.Clear reconstruction. The same was applied for the lower quartile reported in TheraP, using SUVmean ≥ 7 and SUVmean < 7. The patients were subsequently sub-classified into two groups of low and high-volume disease burden using 40 ml cut-off on OSEM reconstructed images [14], and the comparison was done between BPL and OSEM reconstructed images in subgroups of low and high-volume disease. The R software (R Foundation for Statistical Computing, Vienna, Austria) and GraphPad PRISM were used for statistical analysis.

## Results

Fifty patients (mean age: 72.5 years, range: 56–91) with prostate cancer were included in the study. At the time of diagnosis, 52% of the patients had Grade Group 5 prostate cancer. The mean PSA level was 77.3 ng/ml (range: 0.1–889) nearest to the time of scanning.

**Fig. 1:**
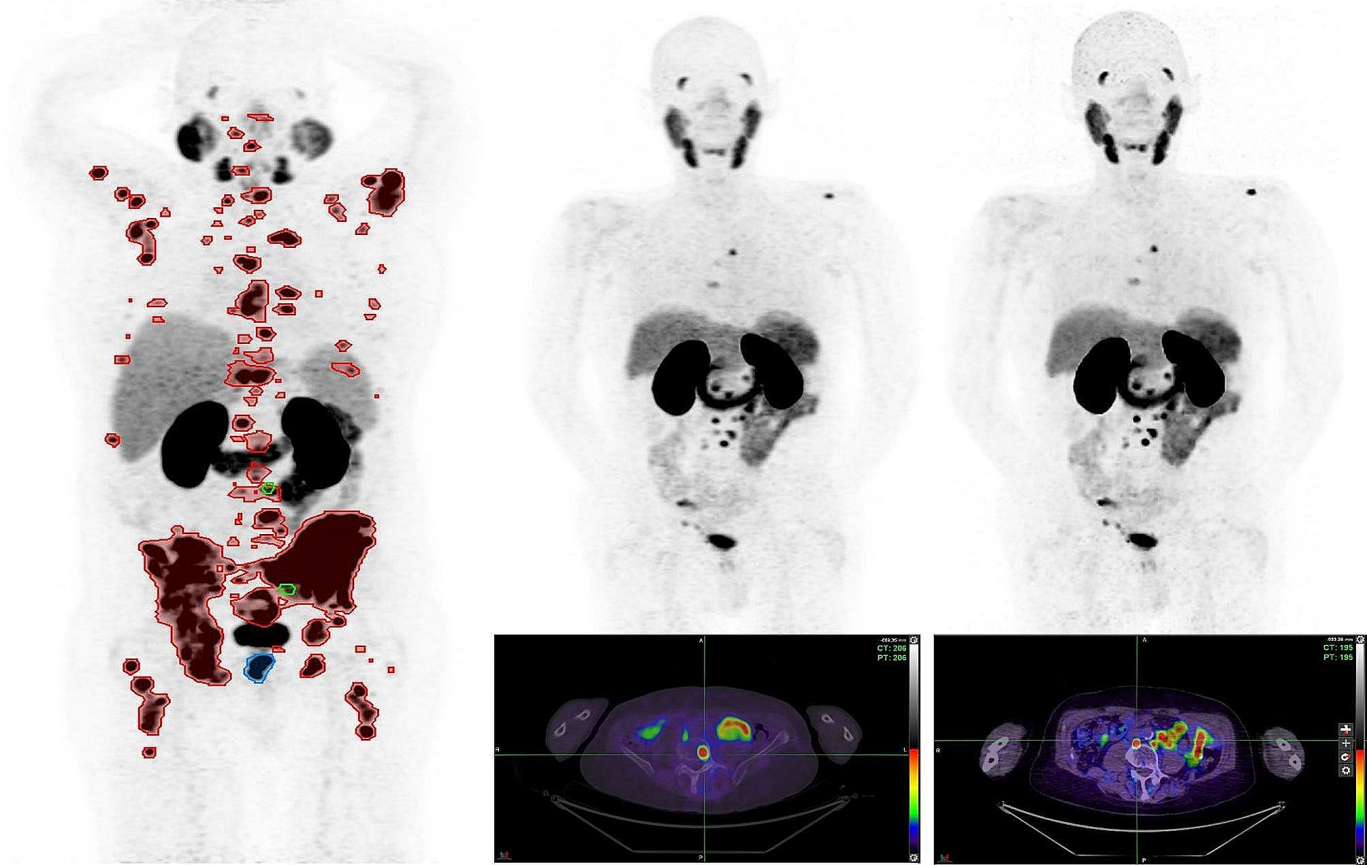
Images quantification (left column) in a patient with local recurrence in prostate bed (blue couture), regional and distant nodal (green contours) and widespread osseous metastatic disease (red contours) with same target lesion (lower thoracic spine) on both OSEM and Q.Clear images. The OSEM (middle column) and BPL (right column) reconstructed images in a different patient, show different target lesions, an osseous lesion (SUVmax: 21.1) on OSEM and a retroperitoneal lymph node (SUVmax: 30.7) on Q.Clear images. The SUVmean and MTV were 6.5 and 55 mL on OSEM compared to 7.1 and 48mL on Q.Clear reconstructed images, respectively

The lesion with the highest SUVmax, also known as the “target lesion” in some trials [1], was an osseous lesion in 40 (80%) and 39 (78%) of patients on OSEM and Q.Clear reconstructed images, respectively. In 10 patients, the target lesion changed from an osseous lesion to another one, and in one patient, the target lesion changed from bone on OSEM to a lymph node lesion on the Q.Clear reconstructed images (Fig. 1). 78% of the patients had high volume disease (> 40 mL) on the OSEM reconstructed images, and no patient was changed from low to high volume disease or vice versa after processing the images using the Q.Clear algorithm (Table 1).

**Table 1:** Comparison between quantitative parameters achieved from OSEM and Q.Clear reconstructed images

	OSEM Recon	Q.Clear Recon
**Target lesion**		
Prostate gland/Prostate bed	3 (6)	3 (6)
Lymph nodes	6 (12)	7 (14)
Bone	40 (80)	39 (78)
Viscera	1 (2)	1 (2)
**Disease volume**		
Low (MTV < 40 mL)	11 (22)	11 (22)
High (MTV > 40 mL)	39 (78)	39 (78)
**Total population**		
SUVmax (range)	38.7 (4.1–133.3)	47.1 (4.4–180.4)
Mean difference (95% CI)		8.4 (5.2–11.5)
SUVmean (range)	7.1 (3.3–13.0)	7.8 (3.3–14.8)
Mean difference (95% CI)		0.7 (0.5–0.9)
MTV (range)	654.5 (2.8–4552.8)	632.9 (2.9–4833.9)
Mean difference (95% CI)		-21.5 (-48.2–26.7)
**Low volume disease (MTV < 40 ml)**		
SUVmax (range)	19.8 (4.1–44.5)	23.8 (4.4–63.4)
Mean difference (95% CI)		4.0 (0.4–7.7)
SUVmean (range)	6.0 (3.3–10.3)	6.6 (3.3–11.8)
Mean difference (95% CI)		0.6 (0.2–1.1)
MTV (range)	15.0 (2.8–24.4)	14.6 (2.9–27.7)
Mean difference (95% CI)		-0.4 (-2.0–1.3)
**High volume disease (MTV > 40 ml)**		
SUVmax (range)	44.1 (9.43–133.3)	53.7 (8.0–180.4)
Mean difference (95% CI)		9.6 (5.7–13.5)
SUVmean (range)	7.4 (4.2–13)	8.1 (4.22–14.8)
Mean difference (95% CI)		0.7 (0.5–0.9)
MTV (range)	834.8 (49–4552.8)	807.3 (48.2–4833.9)
Mean difference (95% CI)		-27.5 (-61.8–6.8)
**SUVmean threshold subgroup analysis**		
SUVmean < 10	39 (78)	37 (74)
SUVmean ≥ 10	11 (22)	13 (26)
SUVmean < 7	25 (50)	24 (48)
SUVmean ≥ 7	25 (50)	26 (52)

Qualitative data are number followed by percentage (*n* = 50); continuous data are mean followed by range

**Fig. 2:**
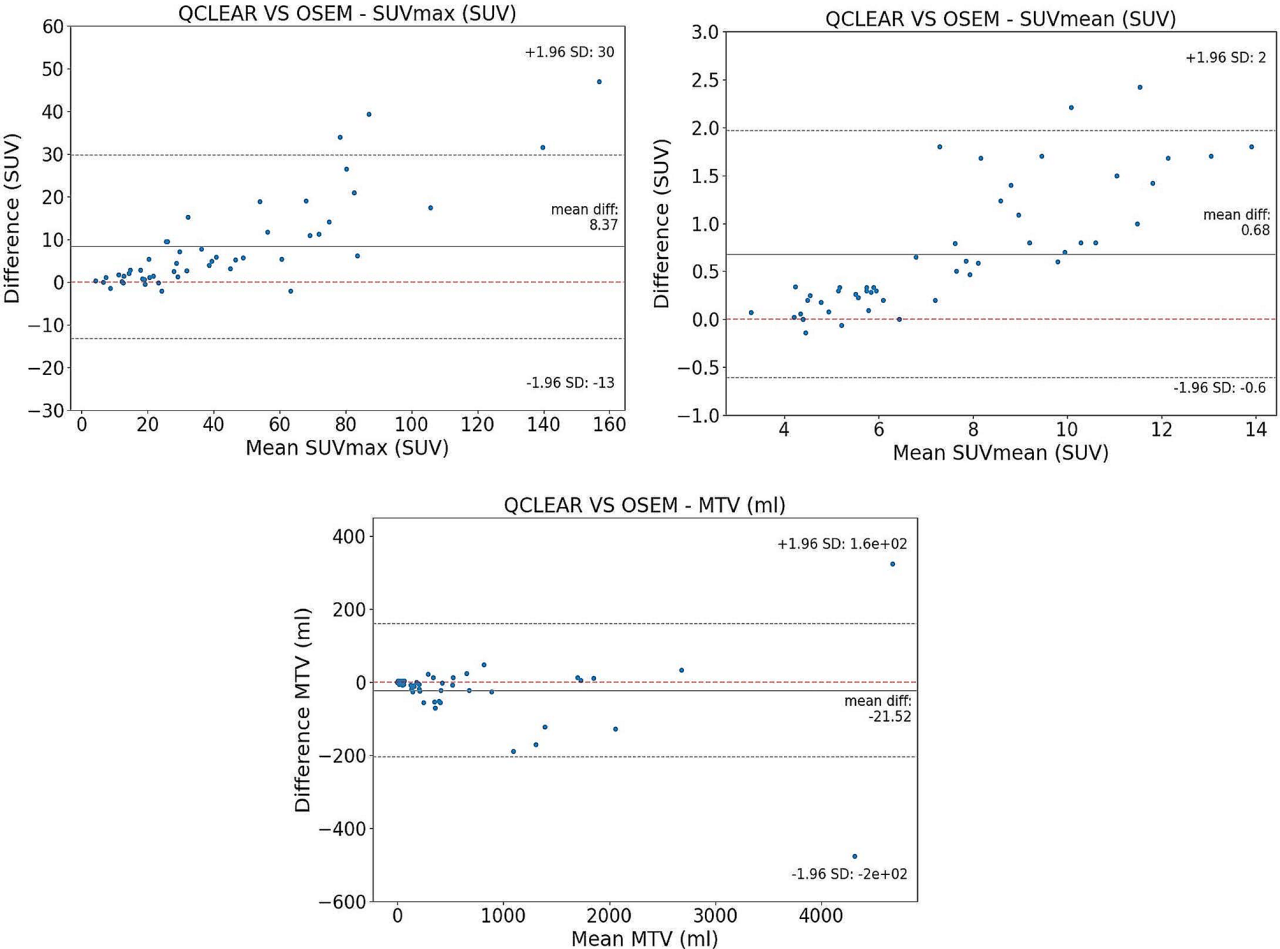
Bland-Altman plots demonstrating the relation between quantitative parameters achieved from OSEM and Q.Clear reconstructed images in SUVmax (left), SUVmean (middle) and MTV (right) in total population (top row) and low (middle row) and high volume (bottom row) subgroups

**Fig. 3:**
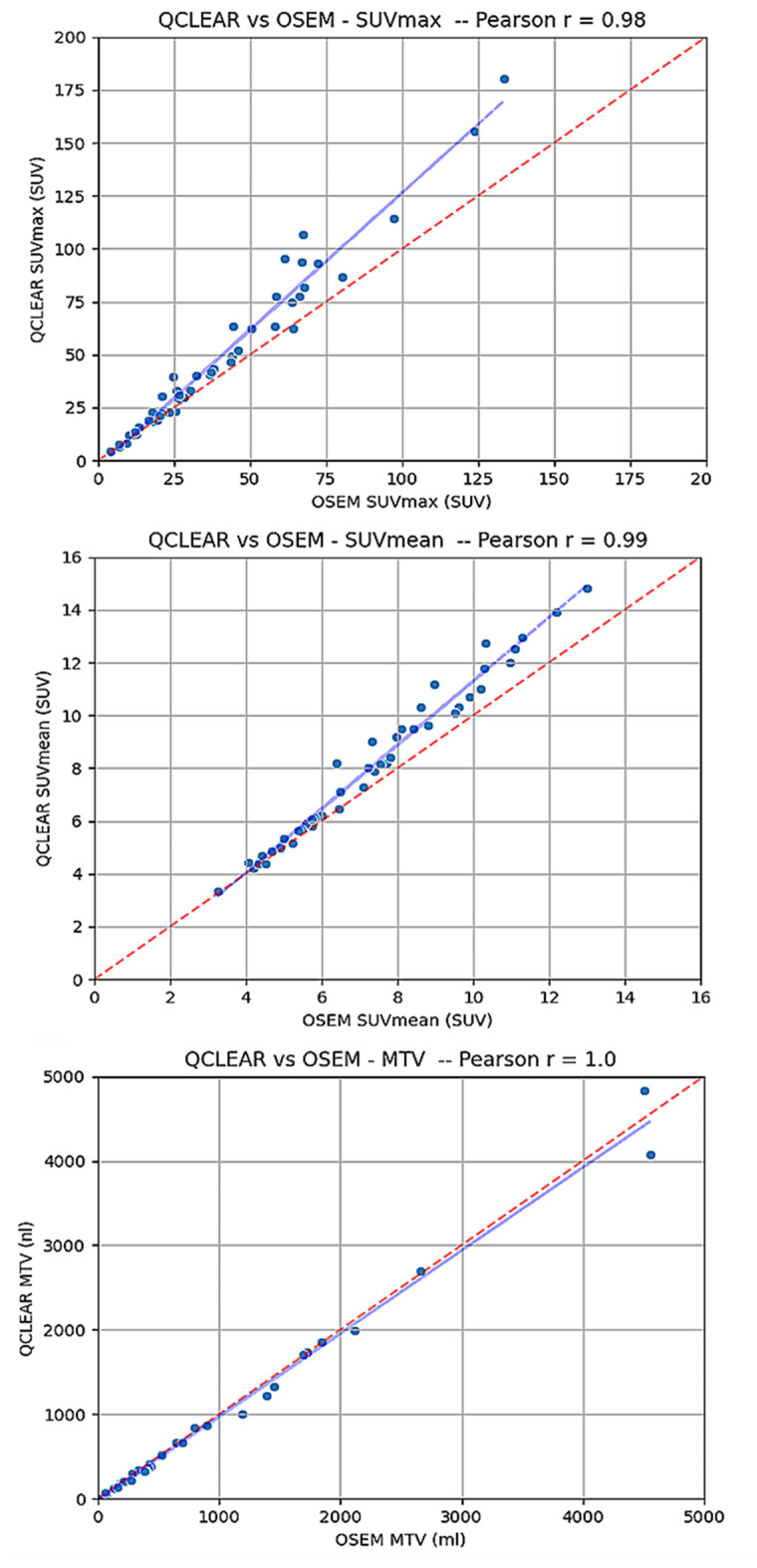
Correlation plots demonstrating the relationship between SUVmax (left plot), SUVmean (middle plot) and MTV (right plot) reported from Q.Clear and OSEM reconstructed images

Using the Bland-Altman plot, the SUVmax and SUVmean were both higher on average with the Q.Clear reconstructed images compared to the OSEM group with a mean difference (95% confidence interval) of 8.4 (5.2–11.5) and 0.7 (0.5–0.9) respectively (Fig. 2). The difference was marked at higher SUVs. The MTV, however, was higher on OSEM compared to Q.Clear reconstructed images with the mean difference of -21.5 (-48.2–26.7). All the parameters demonstrated a strong correlation between OSEM and Q.Clear with Pearson values for SUVmax, SUVmean, and MTV-PSMA of 0.98, 0.99, and 1.0, respectively (Fig. 3).

On subgroup analysis, the Bland-Altman plot showed higher SUVmax on the Q.Clear compared to the OSEM reconstructed images in patients with either low or high volume disease burden with a mean difference of 4.0 (0.4–7.7) and 9.6 (5.7–13.5), respectively. Similarly, higher SUVmean values were observed on Q.Clear reconstructed images in both subgroups (Fig. 2). However, the total tumour volume (MTV-PSMA) was lower on the Q.Clear (807.3 mL) compared to the OSEM reconstructed images (834.8 mL) in patients with high volume disease. The average difference in MTV-PSMA between the OSEM and Q.Clear reconstructed images was − 0.4 mL (-2.0–1.3) in the low volume subgroup (Supplemental Fig. 1).

Using a cut-off value ≥ 10 on SUVmean, 2 patients (4%) were classified differently between the two reconstruction techniques. For the lower threshold of SUVmean ≥ 7, this difference was reduced to 2% of the total population with only one patient changed category (Table 1).

## Discussion

BPL reconstruction (also known as Q.Clear in GE Healthcare Imaging) is a relatively newer reconstruction method with the capacity to improve contrast over OSEM, by applying a noise penalty to individual voxels during reconstruction [8–10]. From a clinical point-of-view, BPL reconstruction improves the signal-to-noise ratio (SNR) and have been reported to be useful for better localization of the tumour as reported in case reports [12, 15]. However, wider adoption of the method showed it cannot increase the sensitivity and specificity of PSMA PET/CT reporting [13], unable to overcome the day-to-day repeatability of the PSMA PET/CT-derived parameters [8] and may even exceeds the EARL-accredited maximum values in phantom studies which may impact development of the PSMA-based response criteria [16]. Moreover, if different reconstruction methods yield to significant variation on the same PET study, it can have downstream consequences leading to variations in the biomarkers and radiomics features derived from the PET study [17].

To quantify PSMA PET/CT, different thresholding methods including histogram-based and predefined fixed thresholding algorithms have been introduced. We chose fixed thresholding of SUV 3 since it was validated prospectively in a multicentre randomised control trial [1]. Our study shows that the differences in SUVmax between OSEM and Q.Clear are non-significant especially in the lower ranges of SUV (Supplemental Fig. 1). This means that alternative use of the Q.Clear method would not change the quantification of whole body PSMA PET metrics by excluding or adding minimally avid lesions (SUV < 3) to the calculated disease burden.

Total tumoral SUVmean on [^68^Ga]Ga-PSMA-11 PET/CT, as a reflector of the average concentration of the radiotracer within the whole tumour burden, has been shown to be a valuable predictive biomarker in patients underwent radionuclide therapy. The TheraP trial showed that SUVmean ≥ 10 was predictive of a higher likelihood for favourable response to [^177^Lu]Lu-PSMA-617 than cabazitaxel with odds ratio of 12.2 vs. 2.2 [3]. Post-hoc analysis of the same study using quartile values of PSMA-PET SUVmean, showed an unfavourable response to radionuclide therapy in subgroup of patients with SUVmean < 6.9 with 29% response to [^177^Lu]Lu-PSMA compared to 43% response to cabazitaxel. Using these two thresholds, our study showed that 2 patients from SUVmean < 10 subgroup on OSEM changed to SUVmean ≥ 10 on Q.Clear while 1 patients from SUVmean < 7 subgroup on OSEM changed to SUVmean ≥ 7 on Q.Clear reconstructed images, with 90% and 96% of patients remaining in the same subgroups, respectively (Table 1). This result supports that Q.Clear reconstruction can accurately differentiate between the patients with lower and the patients with higher likelihood of response to [^177^Lu]Lu-PSMA therapy.

Another important PET parameter is SUVmax which is a frequently used metric to describe lesions due to its simplicity and reproducibility [18]. In a study, BPL reconstructions resulted in significantly higher SUVmax of tumour lesions as compared to standard OSEM reconstructions, with significantly higher relative increases in smaller lesions [8]. This effect emphasised as an area requiring harmonisation in the European Association of Nuclear Medicine with their EARL program [19]. On the advice of the committee, image reconstruction techniques which augment the appearance of small lesions through additional processing may lead to inconsistent quantification of common PET biomarkers across sites or equipment. While our study supports the prior observation of higher average SUVmax on Q.Clear compared to OSEM reconstructed images, it shows a strong correlation between produced SUVmax on these two methods with Pearson r value of 0.98. The correlation plots (Fig. 3) demonstrate the differences in SUV become proportionally more marked at higher SUVs. This is to be expected, given the SNR-recovery of the BPL method as compared to OSEM [12]. Given the proportional difference, harmonising SUVs would be feasible as has been demonstrated in other studies [20]. In addition, the subgroup analysis, did not show significant difference between OSEM and Q.Clear acquired parameters (SUVmax & SUVmean) in subgroup of patients with low-volume disease burden.

Lastly, the MTV-PSMA values were lower in the BPL method as compared to OSEM. This is quite unexpected. Given the fixed absolute value thresholding method (i.e., any voxels with SUV above 3) along with discarding small volumes (i.e., < 0.5 mL) to avoid counting the noise into MTV values, we assume that BPL method spuriously led to small islands with volumes less than < 0.5 which were discarded from the analysis. Apart from this minor difference, true three-dimensional tumour volumetric measurement is also feasible with PET/CT by both methods. Barbato et al. showed that volume quantification with PSMA PET (using a 40 ml cut-off) can discriminatebetween low versus high burden metastatic prostate cancer, with additional sub-classification of disease extension critical for guiding targeted or systemic therapy [14]. The current study showed negligible difference (< 5%) between average MTV-PSMA values by applying these two reconstruction methods in total study population as well as both low and high-volume disease subgroups.

The main clinical implication of these results is the referral to radionuclide therapy centres for consideration of [^177^Lu]Lu-PSMA therapy in patients with BPL reconstructed PSMA PET scans. Although comparative studies should ideally use identical acquisition and processing protocols, this may not always be feasible, particularly in referral centres for radionuclide therapy where new patients with externally-acquired baseline studies are frequently referred. Our results show that the quantified parameters on the PSMA PET studies reconstructed by BPL are reliable for both assessing suitability of [^177^Lu]Lu-PSMA therapy as well as prognostication goals. Therefore, either reconstruction method can be used interchangeably without significant impact on quantified parameters. For centres using BPL reconstruction, it is worth being aware that any differences, although small, are more profound at higher SUVs.

There are some limitations to the current study. The main limitation is using Q.Clear at a single B value, while other centres may use other B values which have not been tested in the current study. Also, the results are only relevant for the GE cameras described, and we cannot extrapolate to newer cameras with digital detectors or manufactures with different algorithms. Finally, there is potential risk of human error in image processing. To minimize this limitation, all lesions contouring was re-evaluated by a second nuclear medicine specialist. To the extent of our knowledge, this is the first study comparing quantified parameters on OSEM and BPL reconstructed [^68^Ga]Ga-PSMA-11 PET scans. The key strength of this study is applying these two reconstruction methods to the same set of patients. This provided the ability to compare same images with completely similar characteristics except for reconstruction method.

## Conclusions

Our study provides evidence that despite higher average SUVmax and SUVmean and lower average MTV-PSMA on Q.Clear reconstruction method compared to OSEM, there is almost perfect correlation between these two reconstruction methods. For SUVmean the differences were very small whereas slightly larger but proportional differences occurred for SUVmax. Accordingly, we conclude there is no significant clinical influence neither on patient selection for [^177^Lu]Lu-PSMA therapy nor on patient prognostication. This study suggests that either reconstruction method can be used clinically; however, for longitudinal comparison, committing to the same reconstruction method would be advisable to minimise variability.

### Electronic supplementary material

Below is the link to the electronic supplementary material.

Supplementary Material 1

## Data Availability

The data is available to be presented upon reasonable request.

## Abbreviations

mCRPC: Metastatic castration-resistance prostate cancer
BPL: Bayesian Penalized Likelihood
OSEM: Ordered Subset Expectation Maximization
SUV: Standardised Uptake Value
MTV: Molecular Tumour Volume
