# Supplementary figures and images for: Comparison of quantitative whole body PET parameters on [68Ga]Ga-PSMA-11 PET/CT using ordered Subset Expectation Maximization (OSEM) vs. bayesian penalized likelihood (BPL) reconstruction algorithms in men with metastatic castration-resistant prostate cancer
Source: Cancer Imaging. 2024 May 6;24:57. doi: 10.1186/s40644-024-00702-x (PMC11075202; doi:10.1186/s40644-024-00702-x)

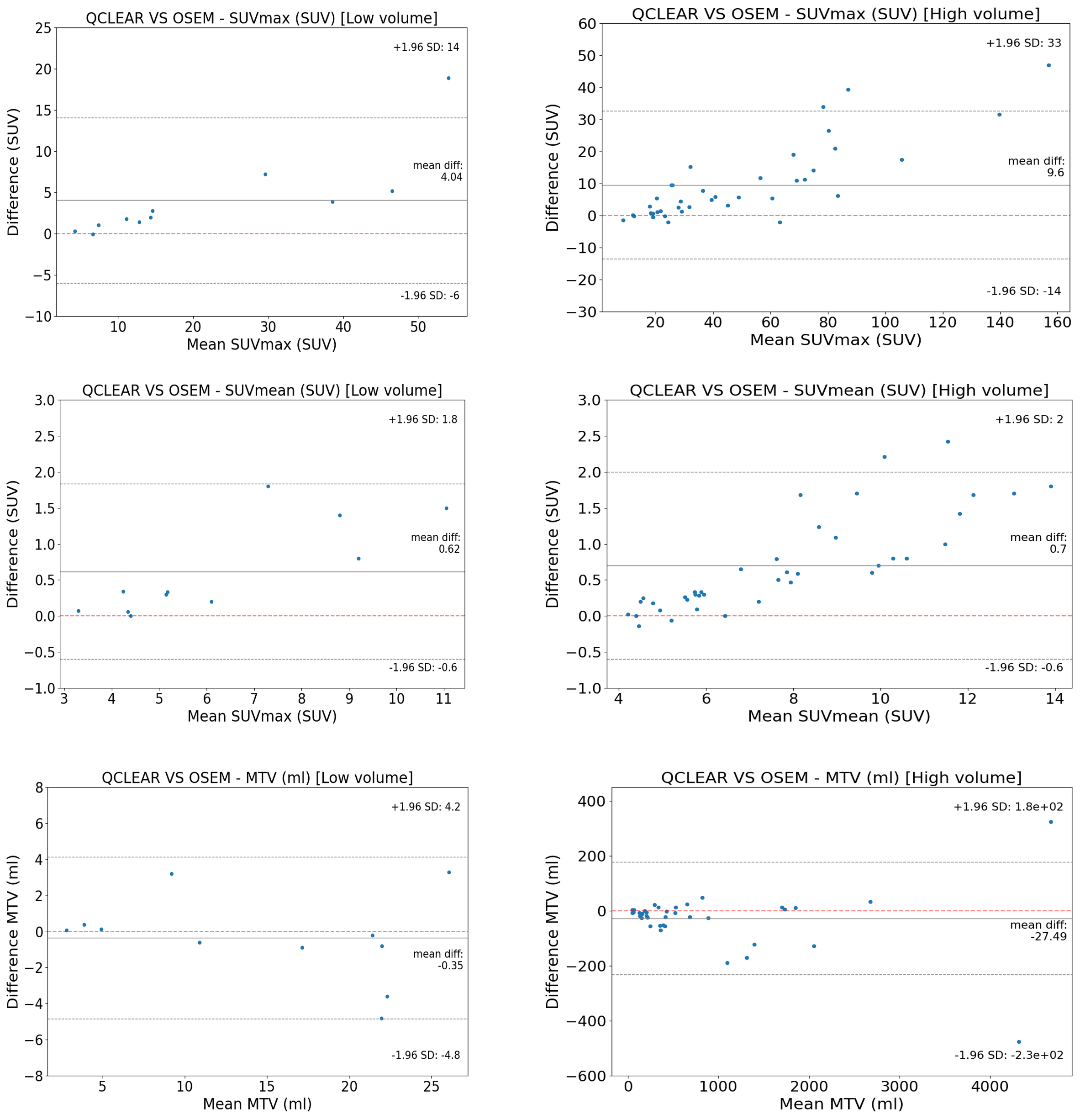

Supplement: Supplementary file 1 — Supplementary Material 1 [file 40644_2024_702_MOESM1_ESM.png]
